# Low-Dose Crizotinib, a Tyrosine Kinase Inhibitor, Highly and Specifically Sensitizes P-Glycoprotein-Overexpressing Chemoresistant Cancer Cells Through Induction of Late Apoptosis *in vivo* and *in vitro*

**DOI:** 10.3389/fonc.2020.00696

**Published:** 2020-05-12

**Authors:** Kyeong Seok Kim, Chunxue Jiang, Ji Young Kim, Jae Hyeon Park, Hae Ri Kim, Su Hyun Lee, Hyung Sik Kim, Sungpil Yoon

**Affiliations:** School of Pharmacy, Sungkyunkwan University, Suwon-si, South Korea

**Keywords:** crizotinib, monotherapy, single treatment, tyrosine kinase inhibitors, cancer, P-gp, drug-resistance

## Abstract

We investigated possible conditions or drugs that could target P-glycoprotein (P-gp)-overexpressing drug-resistant KBV20C cancer cells. Specifically, we focused on identifying a single treatment with a relatively low half maximal inhibitory concentration (IC_50_). Our approach utilized repurposing drugs, which are already used in clinical practice. We evaluated 13 TKIs (gefitinib, imatinib, erlotinib, nilotinib, pazopanib, masatinib, sunitinib, sorafenib, regorafenib, lapatinib, vandetanib, cediranib, and crizotinib) for their sensitizing effects on P-gp-overexpressing drug-resistant KBV20C cells. We found that crizotinib had a much greater sensitization effect than the other tested drugs at relatively low doses. In a detailed quantitative analysis using both lower doses and time-duration treatments, we demonstrated that crizotinib, which increased the levels of apoptosis and G2 arrest, was the best TKI to induce sensitization in P-gp-overexpressing KBV20C cells. Upon comparing resistant KBV20C cells and sensitive KB parent cells, crizotinib was found to markedly sensitize drug-resistant KBV20C cancer cells compared with other TKIs. This suggests that crizotinib is a resistant cancer cell-sensitizing drug that induces apoptosis. In mice bearing xenografted P-gp-overexpressing KBV20C cells, we confirmed that crizotinib significantly reduced tumor growth and weight, without apparent side effects. In addition, although lapatinib and crizotinib have a high P-gp inhibitory activity, we found that co-treatment with crizotinib and vincristine (VIC) did not have much of a sensitization effect on KBV20C cells, whereas lapatinib had a high sensitization effect on VIC-treated KBV20C cells. This suggests that crizotinib is a single-treatment specific drug for resistant cancer cells. These findings provide valuable information regarding the sensitization of drug-resistant cells and indicate that low-dose crizotinib monotherapy may be used in patients with specific P-gp-overexpressing chemoresistant cancer.

## Introduction

Antimitotic drugs inhibit mitosis by targeting microtubules and preventing their polymerization or depolymerization. Paclitaxel, docetaxel, vincristine (VIC), vinorelbine, vinblastine, and eribulin are examples of antimitotic drugs (1–4). Although antimitotic drugs are used widely in cancer treatment, cancer cells may develop resistance to these drugs through various ways.

P-glycoprotein (P-gp) overexpression is a well-known mechanism for antimitotic drug resistance. P-gp is a membrane channel that can pump out antimitotic drugs and thus mitigate drug-induced toxicity in cancer cells (5–8). The identification of sensitization mechanisms or drugs for cancer cells that overexpress P-gp would improve treatment strategies for patients who develop resistance to antimitotic drugs. Although P-gp inhibitors have been developed, their toxicity toward normal cells has resulted in their failure in clinical testing. Therefore, there is a need for novel therapies for P-gp-overexpressing drug-resistant cancers.

In this study, we aimed to identify novel repositioned drugs and their possible application in P-gp-overexpressing drug-resistant cancer cells; we also assessed the increase in the sensitizing efficacy of repositioned drugs when used as a single treatment at a low dose. The urgent need for pharmacological treatments for P-gp-overexpressing resistant cancers may be efficiently addressed if novel treatments using repositioned drugs are identified, because these drugs can be used without further toxicity evaluation. Some repositioned drugs that can sensitize P-gp-overexpressing resistant cancer cells have already been reported, such as aripiprazole, pimozide, azelastine, and nelfinavir (9–15).

Tyrosine kinase inhibitors (TKIs) generally target proteins in the epidermal growth factor receptor (EGFR) family and have been developed as a cancer therapy that prevents growth factor signaling in various cancer models (16–18). They are reversible competitors of ATP for binding at the intracellular catalytic domain of EGFRs. In addition, TKIs act as inhibitors of P-gp in cancer cells (17–23). These drugs have also been reported to induce sensitization in drug-resistant cancer cells (24–27). We also demonstrated that, at a low dose, specific TKIs could sensitize P-gp overexpressing drug-resistant cancer cells when combined with antimitotic drugs (28).

In this study, we compared individual TKIs to identify those with a low half-maximal inhibitory concentration (IC_50_) for drug-resistant KBV20C cancer cells. Through a literature search, we identified 13 TKIs (gefitinib, imatinib, erlotinib, nilotinib, pazopanib, masatinib, sunitinib, sorafenib, regorafenib, lapatinib, vandetanib, cediranib, and crizotinib). We found that a low dose of crizotinib was able to strongly sensitize P-gp-overexpressing drug-resistant KBV20C cells. Furthermore, we investigated the exact mechanisms of crizotinib action *in vitro* and in an *in vivo* xenograft model. As crizotinib is already in clinical use as a targeted anticancer therapy, it may be suitable for the development of therapies for highly drug-resistant tumors.

## Materials and Methods

### Reagents and Cell Culture

Rhodamine123 (Rhodamine) and verapamil were purchased from Sigma-Aldrich (St. Louis, MO, USA). VIC was purchased from Enzo Life Sciences (Farmingdale, NY, USA). Gefitinib, imatinib, erlotinib, nilotinib, pazopanib, masatinib, sunitinib, sorafenib, regorafenib, lapatinib, vandetanib, cediranib, and crizotinib were purchased from Selleckchem (Houston, TX, USA). For *in vivo* xenograft experiments, VIC was purchased from APExBIO technology (TX, USA) and crizotinib was purchased from MedChemExpress (NJ, USA). Aqueous solutions of eribulin (Eisai Korea, Seoul, South Korea) were obtained from the National Cancer Center in South Korea.

Human oral squamous carcinoma cell line, parent sensitive KB, and its multidrug-resistant subline, KBV20C, were obtained from Dr. Yong Kee Kim (College of Pharmacy, Sookmyung Women's University, Seoul, South Korea) and have been previously described (12, 29–31). All cell lines were cultured in RPMI 1640 containing 10% fetal bovine serum, 100 U/ml penicillin, and 100 μg/ml streptomycin (WelGENE, Daegu, South Korea).

### Microscopic Observation

Cells grown in 60-mm diameter dishes were treated with the indicated drugs for 24 or 48 h. The medium was removed, and phosphate-buffered saline (PBS) was added into each dish. Cells were examined immediately in two independent experiments using an ECLIPSE Ts2 inverted routine microscope (Nikon, Tokyo, Japan) with a 4× or a 10× objective lens (Nikon's Microscopy U).

### Rhodamine Uptake Tests

The tests used to assess the ability of a drug to inhibit P-gp were based on a previously described method (14, 15, 32). Briefly, cells grown in 60-mm diameter dishes were treated with the indicated drugs and incubated for 4 h at 37°C. Cells were then incubated with 2 μg/ml rhodamine for 1 h 30 min at 37°C. The medium was removed, and the cells were washed with PBS. The stained cells were analyzed in two independent experiments using a Guava EasyCyte Plus Flow Cytometer (Merck Millipore, Burlington, MA, USA).

### Flow Cytometry Analysis

Flow cytometry analysis was performed as previously described (14, 15, 32). Cells were grown in 60-mm diameter dishes and treated with the indicated drugs for 24 h. The cells were then dislodged by trypsin and pelleted by centrifugation. The pelleted cells were washed thoroughly with PBS, suspended in 75% ethanol for at least 1 h at 20°C, washed with PBS, and re-suspended in a cold propidium iodide (PI) staining solution (100 μg/ml RNase A and 50 μg/ml PI in PBS) for 30 min at 37°C. The stained cells were analyzed in two independent experiments for relative DNA content using a Guava EasyCyte Plus Flow Cytometer (Merck Millipore, Burlington, MA, USA).

### Annexin V Analysis

Annexin V analysis was conducted by using the annexin V-fluorescein isothiocyanate (FITC) staining kit (BD Bioscience, Franklin, NJ, USA) as previously described (13, 33–37). Cells were grown in 60-mm diameter dishes and treated with the indicated drugs for 24 h. The cells were then dislodged by trypsin and pelleted by centrifugation. The pelleted cells were washed with PBS. Cells in 100 μl of binding buffer received 5 μl of Annexin V-FITC and 5 μl of PI and were, then, incubated for 15 min at room temperature. The stained cells were analyzed in two independent experiments using a Guava EasyCyte Plus Flow Cytometer (Merck Millipore, Burlington, MA, USA).

### Cell Viability Assay

Cell proliferation was measured by a colorimetric assay using the EZ-CyTox cell viability assay kit (Daeillab, South Korea) according to the manufacturer's instructions. Briefly, cells grown in wells of 96-well plates were treated with the indicated drugs for 48 h. Then, they were incubated with 10 μl of EZ-CyTox solution for 1–2 h at 37°C. Absorbance at 450 nm was determined immediately using the VERSA MAX Microplate Reader (Molecular Devices Corp., Sunnyvale, CA, USA). All experiments were performed at least in triplicate and repeated twice.

### Tumor Xenograft Model

*In vivo* xenograft experiments were performed with 6-week-old male nude mice weighing ~20 g (BALB-c nu/nu, Japan SLC, Inc., Hamamatsu, Shizuoka, Japan) as previously described (38–41). They were housed under controlled temperature (22 ± 2°C) and a 12 h light/dark cycle in filtered-air laminar-flow cabinets, and handled using aseptic procedures. The institutional animal care committee of Sungkyunkwan University approved the experimental procedure. The resistant KBV20C cells (5 × 10^5^ cells/0.1 ml) or sensitive parent KB cells (5 × 10^5^ cells/0.1 ml) in serum-free medium containing 50% matrigel were injected subcutaneously into the right flank of nude mice. Then VIC, crizotinib, or vehicle was started after tumor volume reached above 150 mm^3^. Prior to drug administration, the mice with KBV20C or KB tumors were then randomized to four groups (*n* = 4 in each group): Group 1 was the vehicle control group; Group 2 was treated with VIC (0.5 mg/kg); and Group 3 was treated with crizotinib (25 mg/kg). VIC (once/week) was injected intraperinoneally (i.p.) and crizotinib (five times/week) was orally administered for 28 days. For the duration of the experiment, the mice were observed for clinical signs, changes in body weight and possible side-effects of the administered drugs. Tumor volume was measured every other day using calipers. Tumor volume (*V*) was calculated using the following standard formula: *V* (mm^3^) = 0.52(*ab*^2^), where *a* is the length and *b* is the width of tumor (42). Body weights were recorded before dosing and at termination. On day 28, mice were sacrificed by carbon dioxide asphyxiation.

### Statistical Analysis

Data are presented as mean ± standard deviation (S.D.) of at least three independent experiments. Statistical analysis was performed by using Student's *t*-test. Results were considered statistically significant compared to those of the control when ^*^p < 0.05 or ^**^p < 0.01.

## Results

### Low-Dose Crizotinib Has a Greater Sensitizing Effect on P-gp Overexpressing Drug-Resistant KBV20C Cancer Cells Than Other TKIs

We aimed to identify repositioned drugs effective for sensitizing resistant cells alone or in combination with other chemotherapeutic drugs. It has been reported that TKIs could target P-gp-overexpressing resistant cancer cells (24–27). Previously, we also found that specific TKIs, at a low-dose, could sensitize P-gp overexpressing drug-resistant cancer cells when combined with antimitotic drugs (28). Considering that TKIs are already in clinical use, they can be readily used as repositioned cancer-sensitizing chemotherapeutics without the need for further toxicity studies after elucidation of their mechanism of action in resistant cancer cells.

Furthermore, we aimed to identify TKIs that could be used as a single treatment at relatively low doses to sensitize resistant cancer cells. Therefore, we performed a detailed evaluation of 13 TKIs (gefitinib, imatinib, erlotinib, nilotinib, pazopanib, masatinib, sunitinib, sorafenib, regorafenib, lapatinib, vandetanib, cediranib, and crizotinib) to determine their sensitizing effects on P-gp-overexpressing drug-resistant KBV20C cells at relatively low doses.

**Figure 1:**
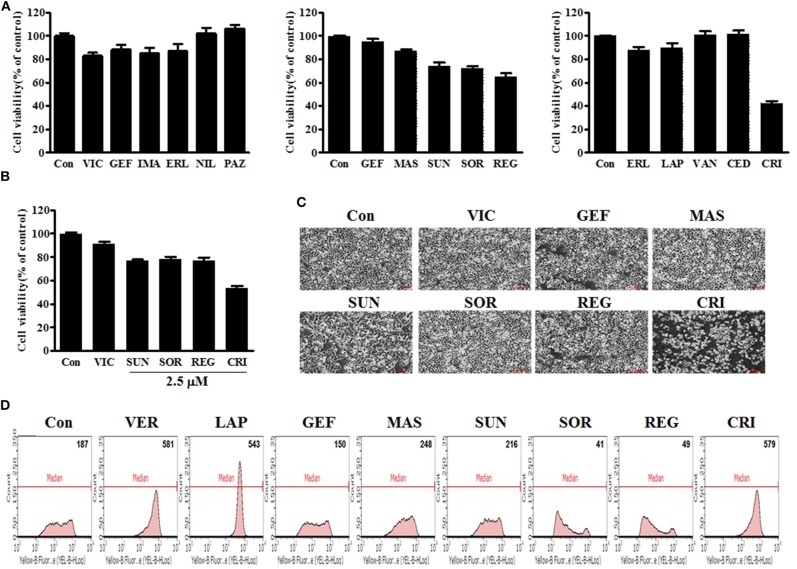
Low-dose crizotinib sensitizes P-gp-overexpressing drug-resistant KBV20C cancer cells to a greater extent than the 12 other TKIs. **(A)** KBV20C cells were plated on 96-well plates and grown to 30–40% confluence. The cells were then stimulated for 48 h with VIC, 5 nM vincristine; GEF, 5 μM of gefitinib; IMA, imatinib; ERL, erlotinib; NIL, nilotinib; PAZ, pazopanib; MAS, masatinib; SUN, sunitinib; SOR, sorafenib; REG, regorafenib; LAP, lapatinib; VAN, vandetanib; CED, cediranib; CRI, crizotinib; or 0.1% DMSO (Con). Cell viability assay was performed as described in “Materials and methods.” The data are presented as the mean ± S.D. of at least in triplicate experiments. **(B)** KBV20C cells were plated on 96-well plates and grown to 30–40% confluence. The cells were then stimulated for 48 h with VIC, 5 nM vincristine; SUN, 2.5 μM of sunitinib; SOR, sorafenib; REG, regorafenib; CRI, crizotinib; or 0.1% DMSO (Con). Cell viability assay was performed as described in “Materials and methods.” **(C)** KBV20C cells were grown on 60 mm-diameter dishes and treated with VIC, 5 nM vincristine; GEF, 5 μM of gefitinib; MAS, masatinib; SUN, sunitinib; SOR, sorafenib; REG, regorafenib; CRI, crizotinib; or 0.1% DMSO (Con). After 1 day, all cells were observed using an inverted microscope at ×4 magnification. **(D)** KBV20C cells were grown on 60 mm-diameter dishes and treated with VER, 10 μM verapamil; LAP, 5 μM of lapatinib; GEF, gefitinib; MAS, masatinib; SUN, sunitinib; SOR, sorafenib; REG, regorafenib; CRI, crizotinib; or 0.1% DMSO (Con). After 4 h, all cells were stained with rhodamine and examined by using Flow cytometry analysis, as described in Materials and Methods.

First, we performed a quantitative analysis of resistant KBV20C-cell viability after they were treated with 5 μM of each TKI being assessed. As shown in Figure 1A, sunitinib, sorafenib, regorafenib, and crizotinib reduced the viability of resistant KBV20C cells, compared with VIC, an antimitotic drug that is routinely used as a chemotherapeutic agent. KBV20C cancer cells exhibit a VIC-resistant phenotype due to P-gp overexpression (13, 15, 29, 30). We also confirmed that sorafenib, regorafenib, and crizotinib have a sensitization effect with a low dose of 2.5 μM (Figure 1B). We found that the viability of crizotinib-treated cells was >50% compared with that of the control (Figures 1A,B), suggesting that crizotinib exerted a stronger sensitizing effect on resistant cancer cells than other TKIs.

As viability tests cannot fully indicate the sensitizing effects of drugs, we confirmed the results of the viability tests by microscopic observation. As shown in Figure 1C, a low dose of crizotinib had a stronger sensitizing effect than other TKIs, as expected. Overall, when we analyzed the sensitizing effect of a single treatment of TKIs on P-gp-overexpressing resistant cancer cells, we observed that low doses of crizotinib had higher sensitization effects than those of other TKIs. Therefore, we concluded that crizotinib was the best drug for sensitizing P-gp-overexpressing drug-resistant cancer cells clinically as a monotherapy with low drug toxicity.

### Only Crizotinib and Lapatinib Have a Strong P-gp-Inhibitory Activity Among all Tested TKIs

In the next phase of our investigation, we evaluated the P-gp-inhibitory activities of TKIs in P-gp-overexpressing KBV20C cells as TKIs have been suggested to sensitize resistant cancer cells with strong P-gp inhibitory activity (27, 28). We also expected that the variation in the degree of P-gp inhibition among TKIs would determine the difference in their sensitizing effects on P-gp-overexpressing drug-resistant KBV20C cancer cells. We tested whether TKIs increase the inhibition of P-gp substrate efflux. Rhodamine 123, a known P-gp substrate, was used to measure P-gp inhibition (12, 37). Green fluorescence within cells was used to quantify the intracellular accumulation of rhodamine 123. The P-gp inhibitor verapamil was used as a positive control (7, 8, 37). We found that crizotinib and lapatinib induced high P-gp inhibitory activity (Figure 1D), whereas other TKIs had very low or no P-gp-inhibitory activity. We found that the proportion of cells was increased by ~3.1-fold after 10 μM verapamil treatment, 2.9-fold after 5 μM lapatinib treatment, and 3.1-fold after 5 μM crizotinib treatment (Figure 1D). As 4 h represent a short treatment duration, we concluded that crizotinib and lapatinib inhibited P-gp by direct binding, similarly to verapamil. Given that previously developed P-gp inhibitors, including verapamil, are toxic to normal cells (5, 7, 8, 37), we believe that crizotinib and lapatinib are adequate alternatives for the sensitization of P-gp-overexpressing resistant cancer cells in clinical settings. Although low doses of sorafenib, regorafenib, and crizotinib have sensitizing effects on the KBV20C cells, only crizotinib does so with a very strong P-gp-inhibitory activity (Figures 1A–D). We also found that lapatinib, which has a strong P-gp inhibitory activity, did not exert a sensitizing effect on P-gp-overexpressing resistant KBV20C cells when applied as a monotherapy (Figures 1A–D), suggesting that sensitization induced by single TKI treatment in KBV20C cells is independent of the P-gp-inhibitory effects of TKIs. Overall, we found that crizotinib and lapatinib strongly inhibited P-gp, but that this was not associated with their sensitizing effect on drug-resistant KBV20C cells when they were used as a monotherapy.

### Crizotinib Exerts Concentration- and Time-Dependent Sensitization in Drug-Resistant KBV20C Cancer Cells

**Figure 2:**
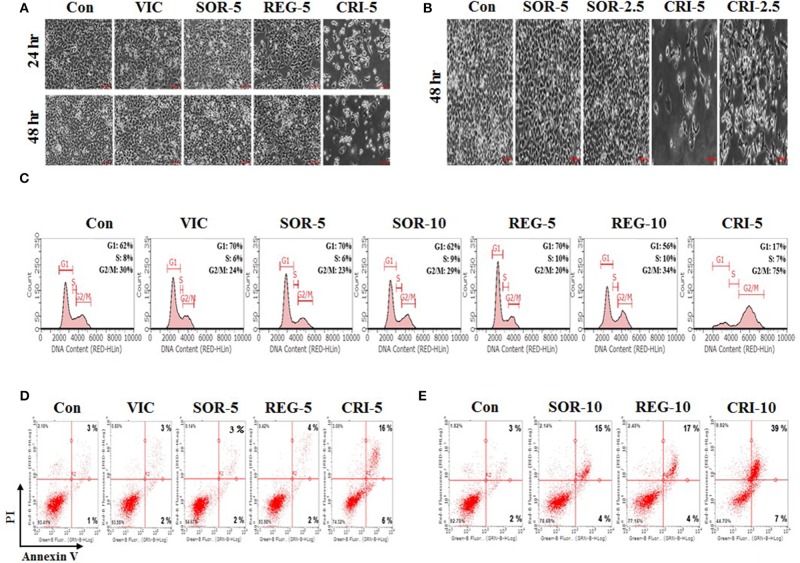
Low-dose crizotinib sensitizes drug-resistant KBV20C cells via apoptosis due to G2 arrest. **(A,B)** KBV20C cells were grown on 60 mm-diameter dishes and treated with VIC, 5 nM vincristine; SOR-2.5, 2.5 μM sorafenib; SOR-5, 5 μM sorafenib; REG-5, 5 μM regorafenib; CRI-2.5, 2.5 μM crizotinib; CRI-5, 5 μM crizotinib; or 0.1% DMSO (Con). After 24 or 48 h, all cells were observed using an inverted microscope at ×10 magnification. **(C)** KBV20C cells were grown on 60 mm-diameter dishes and treated with VIC, 5 nM vincristine; SOR-5, 5 μM sorafenib; SOR-10, 10 μM sorafenib; REG-5, 5 μM regorafenib; REG-10, 10 μM regorafenib; CRI-5, 5 μM crizotinib; or 0.1% DMSO (Con). After 24 h, flowcytometry analyses were performed as described in Materials and Methods. **(D,E)** KBV20C cells were grown on 60 mm-diameter dishes and stimulated with VIC, 5 nM vincristine; SOR-5, 5 μM sorafenib; SOR-10, 10 μM sorafenib; REG-5, 5 μM regorafenib; REG-10, 10 μM regorafenib; CRI-5, 5 μM crizotinib; CRI-10, 10 μM crizotinib; or 0.1% DMSO (Con). After 24 h, Annexin V analyses were performed as described in Materials and Methods.

We tested the sensitization mechanisms of P-gp-overexpressing drug-resistant cancer cells for the following selected drugs: sorafenib, regorafenib, and crizotinib. To observe whether the sensitizing effect of these drugs depended on their concentration and treatment duration, we performed detailed microscopic observations at a higher resolution to assess cellular shape and proliferation rates accurately. As shown in Figures 2A,B, 5 μM crizotinib had a stronger sensitizing effect than 2.5 μM crizotinib, and a treatment period of 48 h resulted in a better sensitizing effect than that of 24 h. This suggests that crizotinib is a suitable drug to sensitize P-gp-overexpressing resistant cancer cells. However, we did not observe any sensitization for either sorafenib or regorafenib following a 5 μM treatment for 48 h (Figures 2A,B), suggesting that higher doses and increased durations of sorafenib and regorafenib are required to exert sensitization in drug-resistant cancer cells. Overall, we identified that only low-dose crizotinib had a concentration- and time-dependent sensitizing effect on P-gp-overexpressing drug-resistant KBV20C cells.

### Low-Dose Crizotinib Strongly Induced G2 Arrest in Drug-Resistant KBV20C Cells

To further clarify the mechanism of action of crizotinib, we performed Flow cytometry analyses (14, 15, 32). As shown in Figure 2C, 5 μM crizotinib considerably increased the number of cells undergoing G2 arrest compared with 10 μM sorafenib and 10 μM regorafenib. We found that the proportion of cells at the G2 arrest stage was ~75% after 5 μM crizotinib treatment, 29% after 10 μM sorafenib treatment, and 34% after 10 μM regorafenib treatment (Figure 2C). As expected, KBV20C drug-resistant cancer cells exhibited a VIC-resistant phenotype in cell cycle arrest due to P-gp overexpression (Figure 2C). The results indicated that low-dose crizotinib induces G2 arrest more strongly than other TKIs. This also indicated that cell cycle arrest induced by crizotinib resulted in a large reduction in cellular viability and proliferation. Overall, we conclude that low-dose crizotinib reduces the viability and proliferation of P-gp overexpressing drug-resistant KBV20C cells via G2 cell cycle arrest.

### Low-Dose Crizotinib Sensitizes Drug-Resistant KBV20C Cells via Apoptosis

Using annexin V analysis, we tested whether crizotinib induces apoptotic cell death. As seen in Figure 2D, apoptotic cell death was greatly increased after treatment with 5 μM crizotinib, compared to treatment with the control or VIC. This indicated that the reduction in the number of cells at the G2 arrest stage by crizotinib contributes to increased apoptotic death. However, despite the induction of cell cycle arrest (Figure 2C), both 5 μM sorafenib and 5 μM regorafenib caused only a slight increase in apoptosis (Figure 2D), suggesting that G2 arrest was highly associated with increased apoptosis in the sensitization of drug-resistant cancer cells by TKIs. In a detailed analysis of annexin V, we determined whether early or late apoptotic cellular death was increased when the drug concentration increased. As seen in Figures 2D,E, crizotinib at 10 μM largely increased late apoptosis in comparison with 5 μM crizotinib, whereas early apoptosis was not increased much. This suggests that crizotinib could directly induce apoptotic cellular death, without delay, via the early apoptotic pathway. In addition, when we increased the concentrations of sorafenib and regorafenib to 10 μM, we found that late apoptosis was notably increased (Figures 2D,E), suggesting that these drugs can sensitize P-gp-overexpressing resistant KBV20C cancer cells at higher doses. Overall, we conclude that low-dose crizotinib strongly sensitized P-gp-overexpressing resistant KBV20C cells via G2 cell cycle arrest and apoptosis.

### Low-Dose Crizotinib Specifically Sensitizes Drug-Resistant KBV20C Cells More Than Drug-Sensitive Parent KB Cells

**Figure 3:**
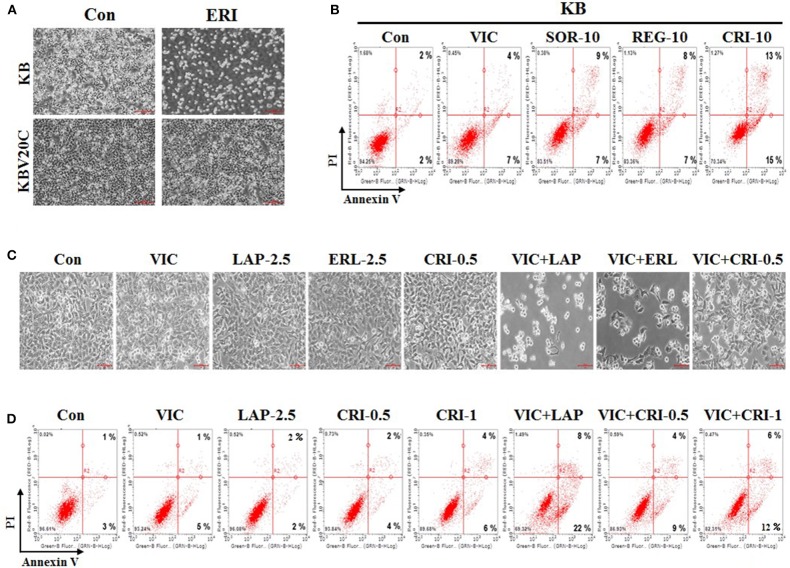
Low-dose crizotinib specifically sensitizes drug-resistant KBV20C cells more than drug-sensitive parent KB cells. **(A)** Parent sensitive KB cells and drug-resistant KBV20C were grown on 60 mm-diameter dishes and treated with 30 nM eribulin (ERI) or 0.1% DMSO (Con). After 1 day, all cells were observed using an inverted microscope at ×4 magnification. **(B)** Parent sensitive KB cells were grown on 60 mm-diameter dishes and stimulated with VIC, 5 nM vincristine; SOR-10, 10 μM sorafenib; REG-10, 10 μM regorafenib; CRI-10, 10 μM crizotinib; or 0.1% DMSO (Con). After 24 h, annexin V analyses were performed as described in Materials and Methods. **(C,D)** KBV20C cells were grown on 60 mm-diameter dishes and stimulated with VIC, 5 nM vincristine; LAP-2.5, 2.5 μM lapatinib; ERL-2.5, 2.5 μM erlotinib; CRI-0.5, 0.5 μM crizotinib; CRI-1, 1 μM crizotinib; VIC+LAP, 5 nM vincristine with 2.5 μM lapatinib; VIC+ERL, 5 nM vincristine with 2.5 μM erlotinib; VIC+CRI-0.5, 5 nM vincristine with 0.5 μM crizotinib; VIC+CRI-1, 5 nM vincristine with 1 μM crizotinib; or 0.1% DMSO (Con). After 24 h all cells were observed using an inverted microscope at ×10 magnification **(C)**, or annexin V analyses were performed as described in Materials and Methods **(D)**.

Next, we compared the sensitizing effect of crizotinib on drug-resistant KBV20C cells and drug-sensitive parent KB cells, as low-dose crizotinib is able to specifically sensitize P-gp-overexpressing drug-resistant KBV20C cells while not affecting drug-sensitive parent KB cells. We previously reported that P-gp-overexpressing KBV20C cells were highly resistant to antimitotic drugs, such as eribulin (12, 36, 37). Eribulin was recently developed and is a promising drug for the treatment of drug-resistant cancer (43–45). As shown in Figure 3A, KBV20C cells are highly eribulin-resistant compared to the parent KB cells.

In this experiment, we compared sorafenib, regorafenib, and crizotinib, which showed greater sensitizing effects toward drug-resistant KBV20C cells compared with other TKIs (Figures 1A–C). As shown in Figure 3B, the percentage of drug-sensitive KB cells at early and late apoptosis stages was 16% for sorafenib, 15% for regorafenib, and 28% for crizotinib. This suggests that a higher proportion of drug-sensitive KB cells underwent apoptotic death after crizotinib treatment than that after sorafenib and regorafenib treatment. However, the percentage of apoptotic drug-resistant KBV20C cells at early and late apoptosis stages was 19% for sorafenib, 21% for regorafenib, and 46% for crizotinib (Figure 2E); low-dose crizotinib was more effective than sorafenib and regorafenib in sensitizing KBV20C cells specifically, while sparing parent KB cells. We therefore concluded that crizotinib has a relatively greater specificity for sensitizing KBV20C cells than KB cells. As expected, drug-sensitive KB cells exhibited a VIC-sensitive phenotype (Figure 3B). In addition, we found that late apoptotic death was specifically increased in drug-resistant KBV20C cells by TKIs. In the detailed quantitative analysis, late apoptosis was found to occur in 9, 8, and 13% of drug-sensitive KB cells after sorafenib, regorafenib, and crizotinib treatment, respectively (Figure 3B), whereas late apoptosis occurred in 15, 17, and 39% of drug-resistant KBV20 cells after sorafenib, regorafenib, and crizotinib treatment, respectively (Figure 2E). This suggests that apoptotic cell death was directly induced in KBV20C cells, without delay, via the arrest of the early apoptotic pathway. However, apoptosis appeared to be delayed in KB cells by the treatments, and a longer duration or higher dose was needed for inducing a sensitizing effect similar to that induced in KBV20C cells in these cells.

Altogether, in our study, which aimed to select a TKI that could be used as a resistant cancer cell-sensitizing monotherapy from among 13 TKIs, we identified low-dose crizotinib to strongly sensitize P-gp-overexpressing antimitotic drug-resistant cancer cells via the induction of late apoptosis and G2 arrest. Notably, low-dose crizotinib had a much stronger sensitizing effect on drug-resistant KBV20C cells than on drug-sensitive KB cells. These findings also provide valuable information on the sensitization of drug-resistant cells and indicate that crizotinib may be used in patients with drug-resistant cancer as a specific monotherapy.

### Crizotinib Was Less Effective Than Other TKIs in VIC Co-treated Resistant KBV20C Cells

We previously demonstrated that when used as a combination therapy, lapatinib and erotinib sensitized VIC-treated KBV20C cells and that they could be used as repositioned drugs (28). We tested whether crizotinib and VIC would be more effective as a co-treatment for drug-resistant KBV20C cells compared with other TKIs. Microscopic observations indicated that VIC-lapatinib and VIC-erlotinib co-treatments showed strong sensitizing effects, whereas the VIC-crizotinib co-treatment only had a small effect (Figure 3C). We also confirmed this through apoptosis analysis using annexin V staining (Figure 3D). Although lower doses of lapatinib and erlotinib can be used as a combination therapy with an antimitotic drug, we concluded that low-dose crizotinib could be useful as a single treatment or monotherapy in drug-resistant cancer.

### Crizotinib Specifically Reduced the Growth of Drug-Resistant KBV20C Tumors in Xenografted Mice

**Figure 4:**
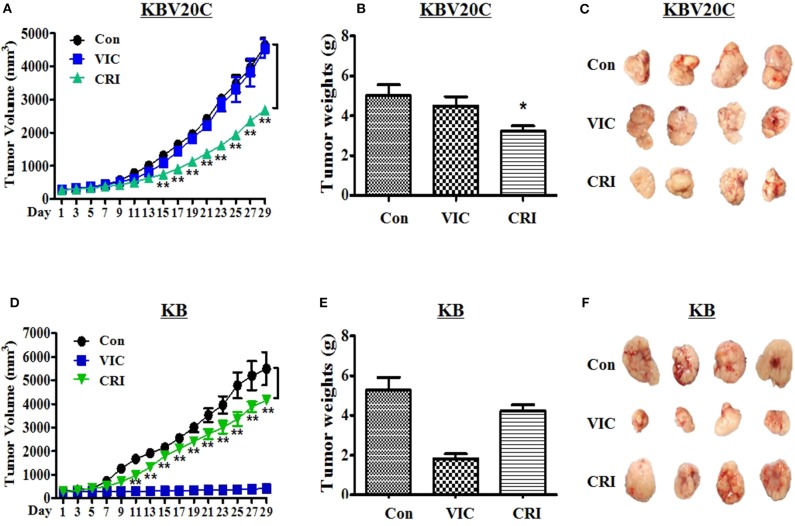
Crizotinib specifically reduces the growth of resistant KBV20C cell tumors in a xenograft model. The experiments were performed as described in Materials and Methods. Mice with established and palpable tumors with KBV20C **(A–C)** or KB **(D–F)** cells were randomized into three groups. Each group with four tumor-bearing mice were administered with vehicle control (Con), vincristine (VIC; 0.5 mg/kg/i.p.), or crizotinib (CRI; 25 mg/kg/p.o). There was no statistically significant difference in body weight of the mice among the treatment groups. **(A,D)** The changes in tumor volume with time after tumor cell implantation. Tumor volume was measured every other day. During the 28-day treatment, tumor volumes were estimated using measurements taken from external calipers (mm cubed). The graph represents tumor size of control, vincristine (VIC)- or crizotinib (CRI)-treated mice. We performed Student's *t*-test to compare control and crizotinib-treated groups. **(B,E)** The weight of tumor was measured at the end of treatment. Mice from each group were sacrificed at 28 days and tumors were isolated from mice at the end of treatment. The weight of tumor was measured at the end of treatment. The weight of each harvested tumor was measured and plotted. We performed Student's *t*-test to compare control and crizotinib-treated groups. Each bar represents the mean of tumor weight. Each data point represents the mean ± SD from four mice. *P < 0.05, **P < 0.01. **(C,F)** Tumors were isolated from the mice at the end of treatment. The photograph of tumor size was taken on the 28th day after implantation. Photographs of representative tumors are shown.

Finally, we aimed to understand the antitumor action of crizotinib in *in vivo* BALB/c nude mouse xenograft models of drug-resistant KBV20C cells and drug-sensitive parent KB cells. We determined detailed procedures of this experiment as previously described studies (38–41). We inoculated the mice with crizotinib followed by VIC (0.5 mg/kg), crizotinib (25 mg/kg), or vehicle-treated control for 4 weeks. As shown in Figures 4A–C, treatment of nude mice with crizotinib markedly inhibited the resistant KBV20C tumor growth compared with vehicle treatment. Detailed analysis showed that crizotinib treatment significantly reduced the tumor volume by 50% (Figure 4A) and tumor weight by ~40% (Figures 4B,C), relative to the control group. These data show that crizotinib significantly inhibited the growth of KBV20C cells *in vivo*.

Furthermore, we tested whether crizotinib showed a lower sensitizing-effect on drug-sensitive parent KB cells in the *in vivo* xenograft model (Figures 2D,E, 3B). As expected, drug-sensitive KB cells showed a VIC treatment time-dependent inhibition of tumor growth compared with the control group, whereas drug-resistant KBV20C cells were not affected by VIC treatment (Figures 4A,D). As seen *in vitro*, the apoptosis assay results differed between drug-sensitive KB cells and drug-resistant KBV20C cells (Figures 2D,E, 3B); we confirmed that crizotinib had a greater effect on KBV20C cells than on KB cells in the *in vivo* xenograft model (Figures 4A–F). This suggests that crizotinib specifically sensitizes drug-resistant cancer cells. In addition, as our involved crizotinib, it was also important to check the toxicity of crizotinib in mice. No signs of other unusual toxic effects were observed during the period, as determined through the regular monitoring of the body weight of crizotinib-treated mice (data not shown). Therefore, we confirmed the *in vivo* cytotoxic mechanism of crizotinib in the KBV20C-tumor xenograft model. Collectively, these data showed that the inhibitory effects of crizotinib on tumor cell proliferation *in vitro* were correlated with the *in vivo* anticancer effects. Our study may help improve crizotinib-based monotherapy for patients with cancer, through the independence of chemotherapeutic treatments.

## Discussion

Drug repurposing, is the application of known drugs for new indications. It has been used for the treatment of various diseases and it is advantageous because of the relatively low cost involved and the reduced time required for toxicity tests (9–11). There is an urgent need for pharmacological treatments for drug-resistant cancers, which may be efficiently addressed by drug repurposing, which can offer drugs that can be administered to patients as soon as their efficacy is proven. In the current study, we investigated a novel application of TKIs as repositioned anticancer drugs for sensitizing P-gp-overexpressing drug-resistant cancer cells. It has been shown that TKIs could enhance the sensitivity of P-gp-overexpressing resistant cancer cells (19–27). Recently, we also found that P-gp-overexpressing KBV20C cancer cells highly resistant to antimitotic drugs were sensitized to them upon co-treatment with relatively low doses of lapatinib, erlotinib, gefitinib, or imatinib (28). However, the effects of low doses of these TKIs as monotherapies have not yet been investigated.

Through a literature search, we found 13 TKIs (gefitinib, imatinib, erlotinib, nilotinib, pazopanib, masatinib, sunitinib, sorafenib, regorafenib, lapatinib, vandetanib, cediranib, and crizotinib) that are already being used in the clinic. We then tested these TKIs with the aim of identifying those that could be used as monotherapies to sensitize P-gp-overexpressing drug-resistant KBV20C cancer cells at low doses and with good specificity. We identified crizotinib to be able to sensitize drug-resistant KBV20C cells at a lower dose than other TKIs. Although sunitinib, sorafenib, and regorafenib monotherapies also showed sensitizing effects on drug-resistant KBV20C cells, a much higher dose was needed for these than that required for crizotinib. Although drug-resistant cancer cell-sensitizing effects of TKIs have been demonstrated, our research suggests the pioneering use of crizotinib monotherapy for targeting P-gp-overexpressing drug-resistant cancers. As our results are the first to reveal that low-dose crizotinib monotherapy can be an effective repositioned-drug monotherapy for P-gp-overexpressing drug-resistant cancer, our findings also suggest a role for crizotinib in the prevention or reduction of drug-resistant cancer in clinic. Considering that the use of chemotherapeutic agents for patients with cancer is strongly correlated with a higher incidence of recurrence of drug-resistant cancer due to P-gp overexpression (4, 5, 7), our findings may also contribute to the selection of crizotinib for the prevention or reduction of the incidence of drug-resistant cancer in early therapy for patients with cancer. For example, crizotinib may be suitable as a first-line treatment for patients with cancer in order to prevent the development of drug-resistant cancer. Further mechanistic studies of crizotinib were thus performed.

A detailed analysis was performed to determine the molecular mechanisms underlying the sensitizing effects of crizotinib so that it may be applied clinically in the near future, especially in patients resistant to monotherapy or first-line therapy, to prevent the occurrence of drug-resistant cancer. Through microscopic, Flow cytometry, and annexin V analyses, we found that apoptosis was increased by crizotinib through increased G2 arrest and reduced proliferation. By a more detailed quantitative Flow cytometry and annexin V analysis, we observed that low-dose crizotinib showed a much higher potential to cause G2 arrest and late apoptotic cellular death. Considering that increased late apoptosis results in more rapid cellular death, we concluded that low-dose crizotinib strongly sensitized P-gp-overexpressing resistant KBV20C cells, without prolonging early apoptosis and causing immediate cell death. In addition, we demonstrated that low-dose crizotinib had concentration- and time-dependent sensitizing effects on P-gp-overexpressing drug-resistant KBV20C cells, suggesting that lower doses and a shorter duration of crizotinib treatment are sufficient to sensitize drug-resistant cancer cells. These results indicated that crizotinib is an ideal monotherapy for drug-resistant cancer. Given that among the 13 TKIs tested only crizotinib showed strong sensitizing effects and induced late apoptosis at a relatively low dose in P-gp-overexpressing resistant KBV20C cells, we believe it can be assumed that the sensitizing ability of a drug is not correlated with its general tyrosine kinase inhibitory function.

As EGFR or growth signaling tyrosine receptor kinases are located in the cellular membrane (16, 18), we hypothesized that TKIs, including crizotinib, participate in modifying the P-gp activity in the membranes of drug-resistant cancer cells. As drug efflux via P-gp is the main mechanism for the drug-resistance of KBV20C cells, we hypothesized that the different sensitization effects of the 13 TKIs resulted from their P-gp inhibitory activity. We demonstrated that only low doses of lapatinib and crizotinib have similar P-gp-inhibitory effects to those of the well-known P-gp inhibitor verapamil, whereas other TKIs have lower P-gp inhibitory effects, similar to control levels. As P-gp inhibitors are toxic to normal cells (5, 7, 8), we believe that lapatinib and crizotinib might be considered as P-gp inhibitors able to sensitize P-gp-overexpressing drug-resistant cancer cells.

However, although we did not observe that lapatinib was not correlated with sensitization of P-gp-overexpressing resistant KBV20C cells, we found that sorafenib, regorafenib, and crizotinib at low doses have sensitization effects as single treatments. These results indicate that the sensitization induced by single treatment with TKIs in KBV20C cells is independent of their P-gp-inhibitory effects, although crizotinib has a strong P-gp inhibitory activity. Collectively, we found that crizotinib and lapatinib had strong P-gp inhibitory effects, which did not correlate with their ability to sensitize drug-resistant KBV20C cells as monotherapies.

Previously, we demonstrated that lapatinib and erotinib as a combination therapy sensitized VIC-treated KBV20C cells and could be used as a repositioned drug (28). As crizotinib has strong P-gp inhibitory activity, we tested whether a low dose of crizotinib can also be used as a combination therapy with antimitotic drugs. We observed that low doses of crizotinib did not have strong sensitizing effects in combination with antimitotic drugs, whereas low doses of other TKIs (lapatinib or erlotinb) had a greatly increased sensitizing effect in combination with antimitotic drugs. We concluded that only low-dose crizotinib was very effective as a monotherapy for P-gp-overexpressing resistant cancer. However, our results indicate that other TKIs (lapatinib, erlotinib, gefitinib, and immatinib) at low doses can be only effective as a combination-therapy against P-gp overexpressing resistant cancer cells, as previously reported (28).

A highlight of our findings is that low-dose crizotinb therapy has a more specific sensitizing effect on P-gp-overexpressing drug-resistant KBV20C cells than on drug-sensitive parent KB cells. Especially, we found that crizotinib specifically and highly increased late apoptotic death in drug-resistant KBV20C cells, compared with drug-sensitive KB cells. This suggests that crizotinib is an ideal drug for KBV20C cells, as it can induce direct apoptotic cellular death, without allowing a chance of survival via arrest in the early apoptotic pathway. Eribulin is a recently developed promising antimitotic drug for the treatment of resistant cancers (43–45). We found that P-gp-overexpressing KBV20C cells were highly resistant to eribulin compared with drug-sensitive KB cells (14, 36, 37), and we assumed that crizotinib could be a useful drug against eribulin-resistant cancer cells, which arise from eribulin chemotherapy.

Finally, our results were confirmed in *in vivo* xenograft models. The weight and volume of tumors treated by crizotinib were highly reduced in the P-gp-overexpressing KBV20C xenograft model. We also confirmed *in vivo*, the *in vitro* results for the specificity of crizotinib toward drug-resistant KBV20C cells compared with that toward drug-sensitive KB cells. The sensitive KB cells were not moved as much as resistant KBV20C cells, suggesting that crizotinib specifically enhanced the toxicity of P-gp-overexpressing drug-resistant cancer. We also confirmed that crizotinib did not affect normal growth of the body or normal tissues, suggesting that toxicity of crizotinib is limited to only drug-resistant cancer cells. Our *in vivo* results can, thus, facilitate more rapid clinical application of crizotinib. In further analysis, our results with crizotinib confirm its specificity for targeting only P-gp-overexpressing cancer cells, using other organ-originated P-gp-overexpressing resistant cancer cells and P-gp-transfected cells. The identification of target molecules is also required for future studies in P-gp-overexpressing resistant cancer cells.

Crizotinib is known to target c-Met or ALK (41, 46–50), whereas other TKIs usually target EGFR or VEGFR. The inhibitory mechanism of crizotinib targets the ATP binding pocket of tyrosine kinases, such as c-Met and ALK (46–48). It is reported that crizotinib also targets Jak/Stat pathway (41, 51). As, among TKIs, crizotinib somehow targets different proteins, it will be interesting to test in the future whether known inhibitors of c-Met, ALK, and Stat5 have similar sensitizing effects or mechanisms of action against P-gp-overexpressing drug-resistant cancer cells. Crizotinib has also been tested for targeting non small cell lung cancer (NSCLC), which has a highly resistant phenotype and one of the poorest patient prognoses (49, 52). As personalized medicine is currently gaining popularity, our findings for crizotinib may contribute to effective prescriptions to prevent further development of drug-resistant cancer in lung cancer patients who have c-MET and ALK mutation phenotypes.

Our findings may also be helpful for establishing the use of crizotinib as a first-line treatment for patients with NSCLC before P-gp-overexpressing drug-resistant cancer cells are produced due to chemotherapy. In our future studies, we will consider testing crizotinib in cancer cell types that are of pulmonary origin.

In conclusion, our results highlight the novel selective sensitization ability of crizotinib monotherapy in P-gp-overexpressing resistant cancer cells. From among 13 TKIs, we selected crizotinib, which had a low IC_50_ for drug-resistant KBV20C cells. Notably, low-dose crizotinib strongly increased sensitization of drug-resistant cancer cells in a specific manner through largely increased late apoptosis and G2 arrest. As the toxicities of crizotinib have already been documented, the drug can be made readily available for patients with cancer. Our results could contribute to the improvement or replacement of various chemotherapeutic agents used as monotherapies for the treatment of patients with cancers that can become resistant to chemotherapeutic drugs via P-gp-overexpression.

## Data Availability Statement

This article contains previously unpublished data. The datasets generated for this study are available on request to the corresponding authors.

## Ethics Statement

The animal study was reviewed and approved by institutional animal care committee of Sungkyunkwan University. All procedures and the reporting were performed with guidelines of institutional animal care committee.

## Author Contributions

KK, CJ, and JK collected the data, contributed data or analysis tools, and wrote the article. JP, HRK, and SL collected the data and contributed data or analysis tools. HSK and SY conceived and designed the analysis, collected the data, contributed data or analysis tools, and wrote the article. All authors read and approved the final manuscript.

## Conflict of Interest

The authors declare that the research was conducted in the absence of any commercial or financial relationships that could be construed as a potential conflict of interest.

## Abbreviations

TKIs: Tyrosine kinase inhibitors
EGFR: Epidermal growth factor receptor
GEF: Gefitinib
IMF: Imatinib
ERL: Erlotinib
NIL: Nilotinib
PAZ: Pazopanib
MAS: Masatinib
SUN: Sunitinib
SOR: Sorafenib
REG: Regorafenib
LAP: Lapatinib
VAN: Vandetanib
CED: Cediranib
CRI: Crizotinib
ERI: Eribulin
VIC: Vincristine
VER: Verapamil
Rhodamine: Rhodamine123
DMSO: Dimethylsulfoxide
IC_50_: Half maximal inhibitory concentration
P-gp: P-glycoprotein
NSCLC: Non small cell lung cancer
JAK: Janus kinase
Stat: Signal transducer and activator of transcription
S.D.: Standard deviation
i.p.: Intraperinoneally
p.o.: Per oral.
